# Non contiguous-finished genome sequence and description of *Enorma massiliensis* gen. nov., sp. nov., a new member of the Family *Coriobacteriaceae*

**DOI:** 10.4056/sigs.3426906

**Published:** 2013-06-06

**Authors:** Ajay Kumar Mishra, Perrine Hugon, Jean-Christophe Lagier, Thi-Tien Nguyen, Carine Couderc, Didier Raoult, Pierre-Edouard Fournier

**Affiliations:** 1 Aix-Marseille Université, URMITE, Marseille, France

**Keywords:** *Enorma massiliensis*, genome, culturomics, taxono-genomics

## Abstract

*Enorma massiliensis* strain phI^T^ is the type strain of *E. massiliensis* gen. nov., sp. nov., the type species of a new genus within the family *Coriobacteriaceae*, *Enorma* gen. nov. This strain, whose genome is described here, was isolated from the fecal flora of a 26-year-old woman suffering from morbid obesity. *E. massiliensis* strain phI^T^ is a Gram-positive, obligately anaerobic bacillus. Here we describe the features of this organism, together with the complete genome sequence and annotation. The 2,280,571 bp long genome (1 chromosome but no plasmid) exhibits a G+C content of 62.0% and contains 1,901 protein-coding and 51 RNA genes, including 3 rRNA genes.

## Introduction

*Enorma massiliensis* strain phI^T^ (= CSUR P183 = DSMZ 25476) is the type strain of *E. massiliensis* gen. nov., sp. nov, which, in turn, is the type species of the genus *Enorma* gen. nov. This bacterium was isolated from the stool of a 26-year-old woman suffering from morbid obesity as part of a culturomics study aimed at individually cultivating all of the bacterial species within human feces [1]. It is a Gram-positive, anaerobic, non-endospore forming, indole-negative, rod-shaped bacillus.

Comprehensive characterization of the human microbiome and its relationship to health and disease is a major challenge in the 21^st^ century [2]. High-throughput sequencing using metagenomic and 16S rRNA-based techniques has significantly accelerated the rate of characterization of the human gut flora [3,4]. However, several drawbacks of the current metagenomic approaches, such as major discrepancies among different studies, reflect biases of the techniques employed. Recently, a renewed interest in diversified culture methods for “non-cultivable” bacteria, notably environmental [5] and human gut species led to the identification of new bacterial taxa [1,6-17]. However, the “gold standard” DNA-DNA hybridization and other sophisticated methods used to classify new bacterial taxa are expensive, time-consuming, lack reproducibility and inter-laboratory comparability and may not be of any routine use in clinical laboratories. As a consequence, we recently proposed a polyphasic approach [6-17] to describe new bacterial taxa, in which the complete genome sequence and MALDI-TOF of the protein spectrum would be used together with their main phenotypic characteristics (habitat, Gram staining, culture and metabolic characteristics and, when applicable, pathogenicity).

Here, we present a summary classification and a set of features for *E. massiliensis* gen. nov., sp. nov. strain phI^T^ (= CSUR P183 = DSMZ 25476) as well as the description of the complete genomic sequencing and annotation. These characteristics support the circumscription of the genus *Enorma* and its type species *E. massiliensis*.

The family *Coriobacteriaceae* was proposed in 1997 [18] and currently comprises the 13 following genera [19]: *Adlercreutzia* [20], *Asaccharobacter* [21], *Atopobium* [22], *Colinsella* [23], *Coriobacterium* [24], *Cryptobacterium* [25], *Denitrobacterium* [26], *Eggerthella* [27], *Entherorhabdus* [28], *Gordonibacter* [29], *Olsenella* [30], *Paraeggerthella* [29] and *Slackia* [27]. These microorganisms are anaerobic Gram-positive, rod-shaped enteric bacteria [25]. Members of family *Coriobacteriaceae* are usually found in the intestinal microbiota of humans or animals and are involved in the stimulation of a major hepatic detoxification activity and endogenous drug metabolism, and are associated with both the hepatic triglyceride, glucose, and glycogen levels [26].

## Classification and features

A stool sample was collected from an obese, 26-year-old woman living in Marseille, France, who suffered from morbid obesity: BMI=48.2 (118.8 kg, 1.57 meter). At the time of stool sample collection she was not a drug user and was not on a diet. The patient gave an informed and signed consent, and the agreement of the local ethics committee of the IFR48 (Marseille, France) was obtained under agreement 09-022. The fecal specimen was preserved at -80°C after collection. Strain phI^T^ (Table 1) was isolated in 2011 by anaerobic cultivation at 37°C on 5% sheep blood-enriched Columbia agar (BioMerieux, Marcy l’Etoile, France), after 4 days of preincubation of the stool sample with thioglycolate broth in an anaerobic blood culture bottle.

**Table 1:** Classification and general features of *Enorma massiliensis* strain phI^T^ according to the MIGS recommendations [31]

**MIGS ID**	**Property**	**Term**	**Evidence code^a^**
	Current classification	Domain: *Bacteria* Phylum: *Actinobacteria* Class: *Actinobacteria* Order: *Coriobacteriales* Family: *Coriobacteriaceae* Genus: *Enorma* Species: *Enorma massiliensis* Type strain: phI^T^	[32] [33] [18] [18,34] [27] TAS IDA IDA
	Gram stain	Positive	IDA
	Cell shape	rod	IDA
	Motility	Non motile	IDA
	Sporulation	non sporulating	IDA
	Temperature range	mesophile	IDA
	Optimum temperature	37°C	IDA
MIGS-6.3	Salinity	unknown	IDA
MIGS-22	Oxygen requirement	anaerobic	IDA
	Carbon source	unknown	NAS
	Energy source	unknown	NAS
MIGS-6	Habitat	human gut	IDA
MIGS-15	Biotic relationship	free living	IDA
MIGS-14	Pathogenicity Biosafety level Isolation	unknown 2 human feces	
MIGS-4	Geographic location	France	IDA
MIGS-5	Sample collection time	January 2011	IDA
MIGS-4.1	Latitude	43.296482	IDA
MIGS-4.1	Longitude	5.36978	IDA
MIGS-4.3	Depth	Surface	IDA
MIGS-4.4	Altitude	0 m above sea level	IDA

Evidence codes – IDA: Inferred from Direct Assay; TAS: Traceable Author Statement (i.e., a direct report exists in the literature); NAS: Non-traceable Author Statement (i.e., not directly observed for the living, isolated sample, but based on a generally accepted property for the species, or anecdotal evidence). These evidence codes are from the Gene Ontology project [35]. If the evidence is IDA, then the property was directly observed for a live isolate by one of the authors or an expert mentioned in the acknowledgements.

**Figure 1:**
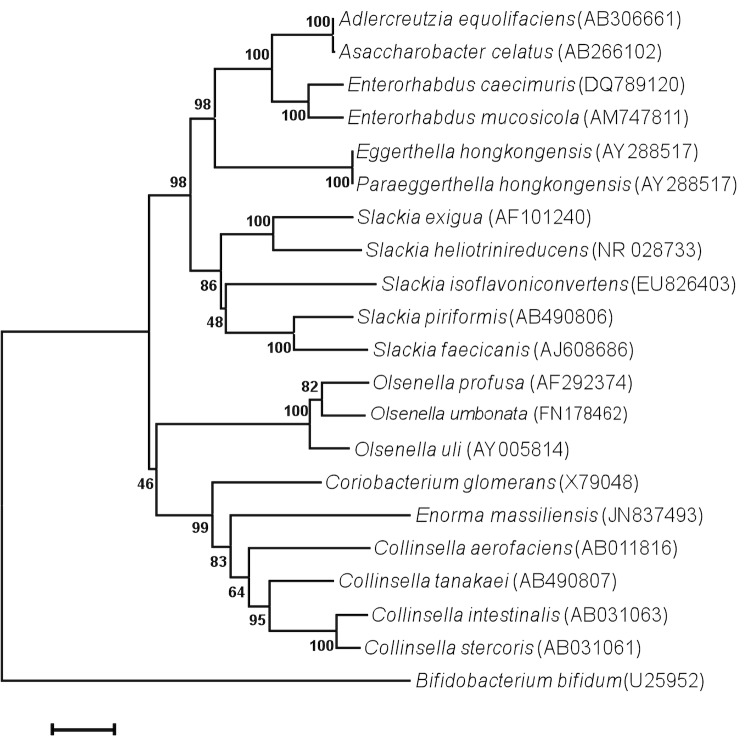
Phylogenetic tree highlighting the position of *Enorma massiliensis* strain phI^T^ relative to other type strains within the *Coriobacteriaceae* family. Genbank accession numbers are indicated in parentheses. Sequences were aligned using CLUSTALW, and phylogenetic inferences obtained using the maximum-likelihood method within the MEGA software. Numbers at the nodes are percentages of bootstrap values obtained by repeating 500 times the analysis to generate a majority consensus tree. *Bifidobacterium bifidum* was used as outgroup. The scale bar represents a 2% nucleotide sequence divergence.

When queried against GenBank, the highest 16S rRNA sequence similarity exhibited by strain phI^T^ was 91.0% when compared to *Collinsella aerofaciens* and *Coriobacterium glomerans*. The organism occupied an intermediate phylogenetic position between these two genera (Figure 1). By comparison with type species of genera from the family *Coriobacteriaceae*, *E. massiliensis* exhibited a 16S rRNA sequence similarity ranging from 84 to 91%. These values are lower than the 95% threshold recommended by Stackebrandt and Ebers [36] to delineate a new genus without carrying out DNA-DNA hybridization, thus suggesting that strain phI^T^ may be classified as a member of a novel genus.

**Figure 2:**
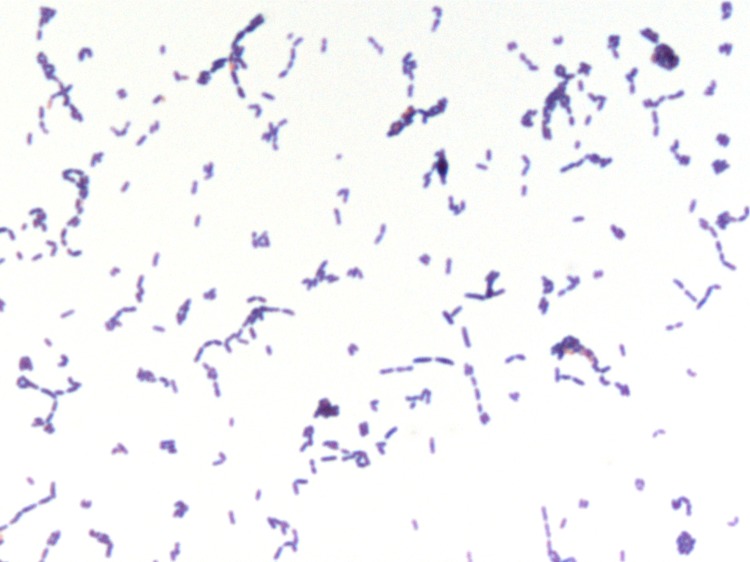
Gram staining of *E massiliensis* strain phI^T^

**Figure 3:**
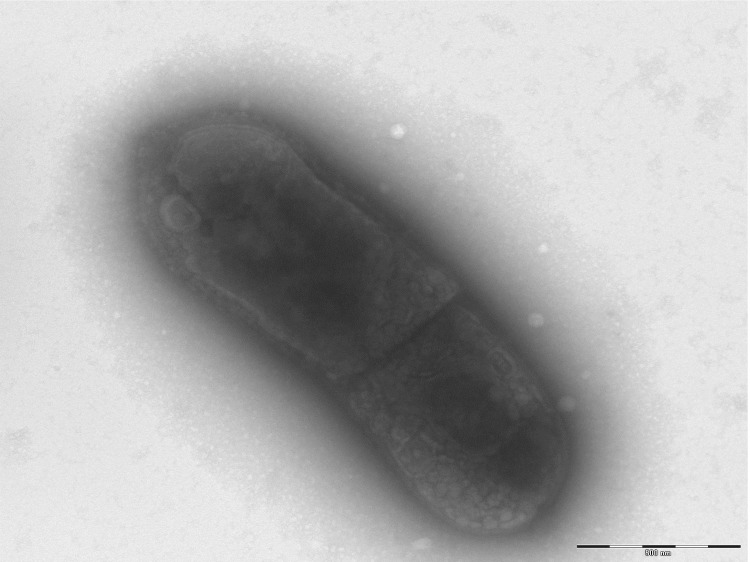
Transmission electron microscopy of *E. massiliensis* strain phI^T^ using a Morgani 268D (Philips) at an operating voltage of 60kV. The scale bar represents 500 nm.

Growth at different growth temperatures (25, 30, 37, 45°C) was tested; no growth occurred at 25°C or 30°C. Growth occurred between 37 and 45°C, but optimal growth was observed at 37°C after 48 hours of incubation. Colonies were light grey and approximately 0.4 mm in diameter on blood-enriched Columbia agar. Growth of the strain was tested in 5% sheep blood-enriched Columbia agar (BioMerieux) under anaerobic and microaerophilic conditions using GENbag anaer and GENbag microaer systems, respectively (BioMerieux), and under aerobic conditions, with or without 5% CO_2_. Growth was achieved only anaerobically. Gram staining showed Gram-positive rods unable to form spores (Figure 2). A motility test was negative. Cells grown on agar are translucent, diameter ranged from 0.50 to 0.64 µm with a mean diameter of 0.57 µm (Figure 3), and length ranged from 0.90 to 1.59 µm with a mean length of 1.19 µm and are mostly grouped in short chains or small clumps.

Strain phI^T^ exhibited neither catalase nor oxidase activities (Table 2). Using an API Rapid ID 32A strip (BioMerieux), positive reactions were observed for α-galactosidase, β-galactosidase, arginine dihydrolase, arginine arylamidase, proline arylamidase, histidine arylamidase, α and β-glucosidase, mannose and raffinose fermentation. Negative reactions were observed for nitrate reduction, indole production, alkaline phosphatase and urease, β-galactosidase 6 phosphate, α-arabinosidase, β-glucuronidase, N-acetyl-β-glucosaminidase, glutamic acid decarboxylase, α-fucosidase, leucyl glycine arylamidase, phenylalanine arylamidase, leucine arylamidase, pyroglutamic acid arylamidase, tyrosin arylamidase, alanine arylamidase, glycine arylamidase, glutamyl glutamic acid arylamidase, and serine arylamidase. Using an API 50CH, no fermentation or assimilation were observed. *E. massiliensis* is susceptible to amoxicillin, amoxicillin-clavulanic acid, metronidazole, imipenem, vancomycin, nitrofurantoin, rifampicin, gentamicin and resistant to penicillin, ceftriaxon, erythromycin, doxycycline, ciprofloxacin and trimethoprim/sulfamethoxazole. By comparison with *C.aerofaciens*, *E.massiliensis* differed in α-galactosidase, β-glucosidase, leucyl glycine arylamidase and glycine arylamidase. By comparison with *C. tanakaei* , *E. massiliensis* differed in alkaline phosphatase, α-galactosidase, β-galactosidase, β-glucuronidase, α-glucosidase, leucyl glycine arylamidase and glycine arylamidase. By comparison with *C. intestinalis*, *E. massiliensis* differed in, alkaline phosphatase, α-and β-galactosidase, α-and β-glucosidase, N-acetyl-β-glucosaminidase, 6-phospho-β-galactosidase, leucyl glycine arylamidase, proline arylamidase and glycine arylamidase.

**Table 2:** Differential characteristics of *Enorma massiliensis* phI^T^, *Collinsella aerofaciens* strain YI^T^ 10235^T^*,* *Collinsella tanakaei* strain YI^T^ 12064^T^, *Coriobacterium glomerans* *strain *PW2 and *Collinsella intestinalis* strain JCM 10643^T^.

**Properties**	*E. massiliensis*	*C. aerofaciens*	*C. tanakaei*	*C.* *glomerans*	*C. intestinalis*
Cell diameter (μm)	0.57	1.2-4.3	0.5-1.0	na	0.3-0.5
Oxygen requirement	anaerobic	anaerobic	anaerobic	anaerobic	anaerobic
Gram stain	+	+	+	+	+
Salt requirement	na	na	na	na	na
Motility	-	na	-	-	-
Endospore formation	-	-	na	-	-
**Production of**					
Alkaline phosphatase	-	-	+	na	+
Acid phosphatase	na	-	+	na	+
Catalase	-	na	-	na	na
Oxidase	-	na	-	na	na
Nitrate reductase	-	na	-	na	na
Urease	-	-	-	na	-
α-galactosidase	+	-	-	na	-
β-galactosidase	+	+	-	na	-
β-glucuronidase	-	-	+	na	-
α -glucosidase	+	+	-	na	-
β-glucosidase	+	-	+	na	var
Esterase	na	-	-	na	-
Esterase lipase	na	-	-	na	-
Indole	-	na	-	na	na
N-acetyl-β-glucosaminidase	-	-	-	na	+
6-Phospho-β -galactosidase	-	-	-	na	+
Argininearylamidase	+	+	+	na	+
Glutamic acid decarboxylase	-	-	-	na	-
Leucyl glycine arylamidase	-	+	+	na	+
Alanine arylamidase	-	-	-	na	-
Proline arylamidase	+	+	+	na	-
Serine arylamidase	-	-	-	na	-
Tyrosine arylamidase	-	-	-	na	-
Glycine arylamidase	-	+	+	na	+
**Utilization of**				na	
D-mannose	+	+	+	na	+
**Habitat**	human gut	human gut	human gut	na	human gut
					

var: variable

w: weak

na: data not available

+/-: depending on tests used

**Figure 4:**
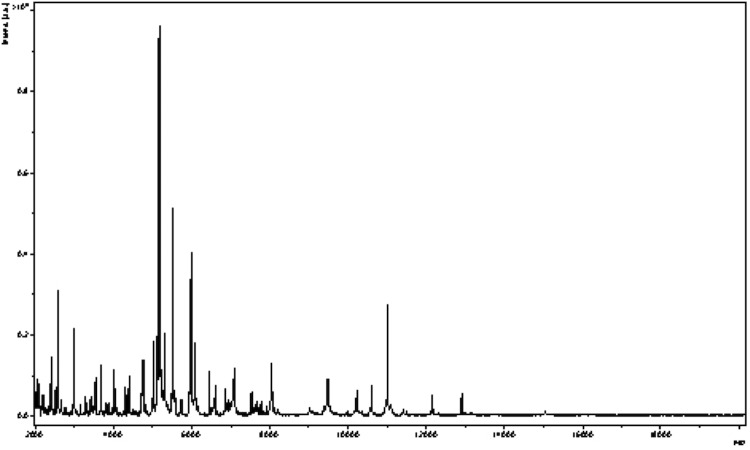
Reference mass spectrum from *E. massiliensis* strain phI^T^. Spectra from 12 individual colonies were compared and a reference spectrum was generated.

**Figure 5:**
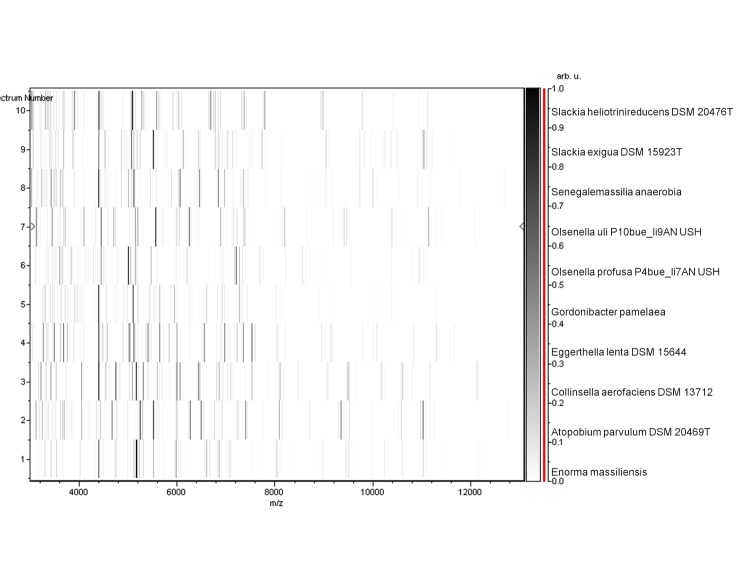
Gel view comparing *Enorma massilienis* phI^T^ spectra with other members of the family *Coriobacteriaceae* (*Slackia heliotrinireducens*, *Slackia exigua*, *Senegalemassilia anaerobia*, *Olsenella uli*, *Olsenella profusa*, *Gordonibacter pamelaea*, *Eggerthella lenta*, *Collinsella aerofaciens* and *Atopobium parvulum*. The Gel View displays the raw spectra of all loaded spectrum files arranged in a pseudo-gel like look. The x-axis records the m/z value. The left y-axis displays the running spectrum number originating from subsequent spectra loading. The peak intensity is expressed by a Gray scale scheme code. The color bar and the right y-axis indicate the relation between the color a peak is displayed with and the peak intensity in arbitrary units.

Matrix-assisted laser-desorption/ionization time-of-flight (MALDI-TOF) MS protein analysis was carried out as previously described [37]. Briefly, a pipette tip was used to pick one isolated bacterial colony from a culture agar plate, and to spread it as a thin film on a MTP 384 MALDI-TOF target plate (Bruker Daltonics, Leipzig, Germany). Twelve distinct deposits were prepared for strain phI^T^ from twelve isolated colonies. Each smear was overlaid with 2µL of matrix solution (saturated solution of alpha-cyano-4-hydroxycinnamic acid) in 50% acetonitrile, 2.5% tri-fluoracetic-acid, and allowed to dry for five minutes. Measurements were performed with a Microflex spectrometer (Bruker). Spectra were recorded in the positive linear mode for the mass range of 2,000 to 20,000 Da (parameter settings: ion source 1 (IS1), 20 kV; IS2, 18.5 kV; lens, 7 kV). A spectrum was obtained after 675 shots at variable laser power. The time of acquisition was between 30 seconds and 1 minute per spot. The twelve phI^T^ spectra were imported into the MALDI BioTyper software (version 2.0, Bruker) and analyzed by standard pattern matching (with default parameter settings) against the main spectra of 3,769 bacteria, which were used as reference data in the BioTyper database. The method of identification included the m/z from 3,000 to 15,000 Da. For every spectrum, 100 peaks at most were taken into account and compared with spectra in the database. A score enabled the identification, or not, from the tested species: a score > 2 with a validly published species enabled the identification at the species level, a score > 1.7 but < 2 enabled the identification at the genus level; and a score < 1.7 did not enable any identification. For strain phI^T^, no significant score was obtained, thus suggesting that our isolate was not a member of a known species. We incremented our database with the spectrum from strain phI^T^ (Figure 4). Finally, the gel view highlighted the spectral differences with other members of the family *Coriobacteriaceae* (Figure 5).

## Genome sequencing information

### Genome project history

The organism was selected for sequencing on the basis of its phylogenetic position and 16S rRNA similarity to other members of the family *Coriobacteriaceae*, and is part of a study of the human digestive flora aiming at isolating all bacterial species within human feces. It was the seventh genome of a *Coriobacteriaceae* and the first genome of *Enorma massiliensis* gen. nov., sp. nov. A summary of the project information is shown in Table 3. The Genbank accession number is CAGZ00000000 and consists of 35 contigs. Table 3 shows the project information and its association with MIGS version 2.0 compliance [31].

**Table 3:** Project information

**MIGS ID**	**Property**	**Term**
MIGS-31	Finishing quality	High-quality draft
MIGS-28	Libraries used	One paired-end 454 3-kb library
MIGS-29	Sequencing platforms	454 GS FLX Titanium
MIGS-31.2	Fold coverage	23
MIGS-30	Assemblers	Newbler version 2.5.3
MIGS-32	Gene calling method	Prodigal
	INSDC ID	PRJEA82083
	Genbank ID	CAGZ00000000
	Genbank Date of Release	May 30, 2012
MIGS-13	Project relevance	Study of the human gut microbiome

### Growth conditions and DNA isolation

*E. massiliensis* gen. nov., sp. nov. strain phI^T^ (= CSUR P183 = DSMZ 25476), was grown anaerobically on 5% sheep blood-enriched Columbia agar (BioMerieux) at 37°C. Four petri dishes were spread and resuspended in 4×100µl of TE buffer and stored at 80°C. Then, 500µl of this suspension was thawed, centrifuged for 3 minutes at 10,000 rpm and resuspended in 4x100µL of G2 buffer (EZ1 DNA Tissue kit, Qiagen). A first mechanical lysis was performed by glass powder on the Fastprep-24 device (Sample Preparation system, MP Biomedicals, USA) using 2×20 seconds cycles. DNA was then treated with 2.5µg/µL lysozyme (30 minutes at 37°C) and extracted using the BioRobot EZ1 Advanced XL (Qiagen). The DNA was then concentrated and purified using the Qiamp kit (Qiagen). The yield and the concentration was measured by the Quant-it Picogreen kit (Invitrogen) on the Genios Tecan fluorometer at 78.9 ng/µl.

### Genome sequencing and assembly

DNA (5 µg) was mechanically fragmented on a Hydroshear device (Digilab, Holliston, MA, USA) with an enrichment size at 3-4kb. The DNA fragmentation was visualized through an Agilent 2100 BioAnalyzer on a DNA labchip 7500 with an optimum size of 3.457kb. A 3kb paired-end library was constructed according to the 454 GS FLX Titanium paired-end protocol (Roche). Circularization and nebulization were performed and generated a pattern with an optimal at 458 bp. After PCR amplification through 15 cycles followed by double size selection, the single stranded paired-end library was then quantified on the Quant-it Ribogreen kit (Invitrogen) on the Genios Tecan fluorometer at 360 pg/µL. The library concentration equivalence was calculated as 1.44E+08 molecules/µL. The library was stored at -20°C until further use.

The paired-end library was amplified with 0.5 cpb in 2 SV-emPCR reactions with the GS Titanium SV emPCR Kit (Lib-L) v2 (Roche). The yield of the emPCR was 20.76%, in the range of 5 to 20% from the Roche procedure.

Approximately 790,000 beads were loaded on 1/4 region of a GS Titanium PicoTiterPlate PTP Kit 70x75 and sequenced with the GS FLX Titanium Sequencing Kit XLR70 (Roche). The run was performed overnight and then analyzed on the cluster through the gsRunBrowser and Newbler assembler (Roche). A total of 237,780 passed filter wells were obtained and generated 52.3Mb with a length average of 220 bp. The globally passed filter sequences were assembled using Newbler with 90% identity and 40 bp as overlap. The final assembly identified 5 scaffolds and 32 large contigs (>1500bp), generating a genome size of 2.28 Mb.

### Genome annotation

Open reading frames (ORFs) were predicted using Prodigal [38] with default parameters but the predicted ORFs were excluded if they spanned a sequencing gap region. The predicted bacterial protein sequences were searched against the GenBank database [39] and the Clusters of Orthologous Groups (COG) databases using BLASTP. The tRNAScanSE tool [40] was used to find tRNA genes, whereas ribosomal RNAs were found by using RNAmmer [41] and BLASTN against the GenBank database. Signal peptides and numbers of transmembrane helices were predicted using SignalP [42] and TMHMM [43] respectively. ORFans were identified if their BLASTP *E*-value was lower than 1e-03 for alignment length greater than 80 amino acids. If alignment lengths were smaller than 80 amino acids, we used an *E*-value of 1e-05. Such parameter thresholds have already been used in previous works to define ORFans. To estimate the mean level of nucleotide sequence similarity at the genome level between *E. massiliensis* strain phI^T^ and other members of *Coriobacteriaceae* family, we compared genomes two by two and determined the mean percentage of nucleotide sequence identity among orthologous ORFs using BLASTn. Orthologous genes were detected using the Proteinortho software [44]. We compared *E. massiliensis* strain phI^T^ with *Collinsella aerofaciens* strain ATCC 25986 (GenBank accession number AAVN00000000), *Collinsella tanakaei* strain YIT 12063 (ADLS00000000) and *Coriobacterium glomerans* strain PW2 (NC_015389). Artemis [45] was used for data management and DNA Plotter [46] was used for visualization of genomic features. The Mauve alignment tool was used for multiple genomic sequence alignment and visualization [47].

## Genome properties

**Figure 6:**
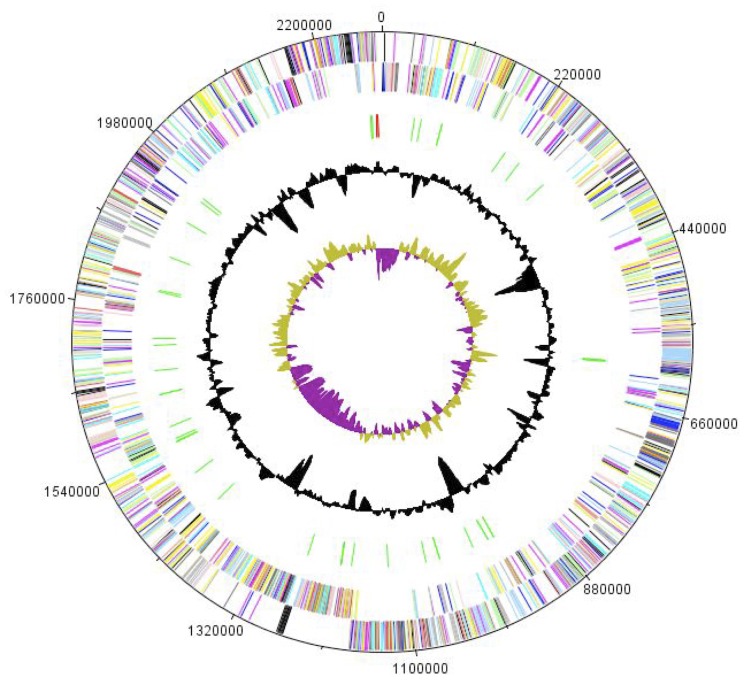
Graphical circular map of the chromosome. From the outside to the inside: open reading frames oriented in the forward (colored by COG categories) direction, open reading frames oriented in the reverse (colored by COG categories) direction, genes on the reverse strand (colored by COG categories), rRNA operon (red) and tRNAs (green), G+C content plot, GC skew (purple: negative values, olive: positive values).

The genome is 2,280,571 bp long (1 chromosome, but no plasmid) with a 62.0% G+C content (Figure 6 and Table 4). Of the 1,952 predicted genes, 1,901 were protein-coding genes and 51 were RNAs, including a complete rRNA operon and 48 tRNAs. A total of 1,486 genes (76.12%) were assigned a putative function. ORFans accounted for 146 of the genes (7.68%). The remaining genes were annotated as hypothetical proteins. The distribution of genes into COGs functional categories is presented in Table 5. The properties and the statistics of the genome are summarized in Tables 4 and 5.

**Table 4:** Nucleotide content and gene count levels of the genome

**Attribute**	Value	% of total^a^
Genome size (bp)	2,280,571	
DNA coding region (bp)	1,9363,32	84.90
DNA G+C content (bp)	1,4139,54	62.0
Coding region (bp)	1,9363,32	84.90
Number of replicons	1	
Extrachromosomal elements	0	
Total genes	1,952	100
RNA genes	51	2.61
rRNA operons	1	
Protein-coding genes	1,901	97.38
Genes with function prediction	1,670	85.55
Genes assigned to COGs	1,486	76.12
Genes with peptide signals	79	4.05
Genes with transmembrane helices	466	23.87
CRISPR repeats	0	

^a^ The total is based on either the size of the genome in base pairs or the total number of protein coding genes in the annotated genome

**Table 5:** Number of genes associated with the 25 general COG functional categories

**Code**	**Value**	**% of total**	**Description**
J	141	7.42	Translation
A	0	0	RNA processing and modification
K	161	8.47	Transcription
L	102	5.37	Replication, recombination and repair
B	1	0.05	Chromatin structure and dynamics
D	15	0.79	Cell cycle control, mitosis and meiosis
Y	0	0	Nuclear structure
V	52	2.74	Defense mechanisms
T	68	3.58	Signal transduction mechanisms
M	90	4.73	Cell wall/membrane biogenesis
N	1	0.05	Cell motility
Z	0	0	Cytoskeleton
W	0	0	Extracellular structures
U	16	0.84	Intracellular trafficking and secretion
O	43	2.26	Posttranslational modification, protein turnover, chaperones
C	73	3.84	Energy production and conversion
G	160	8.42	Carbohydrate transport and metabolism
E	163	8.57	Amino acid transport and metabolism
F	52	2.74	Nucleotide transport and metabolism
H	40	2.10	Coenzyme transport and metabolism
I	38	2.00	Lipid transport and metabolism
P	96	5.05	Inorganic ion transport and metabolism
Q	20	1.05	Secondary metabolites biosynthesis, transport and catabolism
R	225	11.84	General function prediction only
S	113	5.94	Function unknown
-	415	21.83	Not in COGs

a) The total is based on the total number of protein coding genes in the annotated genome.

## Comparison with other *Collinsella* and *Coriobacterium* species genomes

We compared the genome of *E. massiliensis* strain phI^T^ with those of *Collinsella aerofaciens* strain ATCC 25986, *Collinsella tanakaei* strain YIT 12063 and *Coriobacterium glomerans* strain PW2.

The draft genome sequence of *E. massiliensis* strain phI^T^ has a larger size to that of *C. glomerans* genome (2.28 and 2.11 Mb, respectively), but a smaller size than those of *C. aerofaciens* and *C. tanakaei* (2.43 and 2.48 Mb, respectively). The G+C content of *E. massiliensis* is larger than those of *C. glomerans*, *C. aerofaciens* and *C. tanakaei* (62.0, 60.39, 60.55 and 60.29%, respectively).

The gene content of *E. massiliensis* is greater than that of *C. glomerans* (1,901 and 1,768, respectively) but less than that of *C. aerofaciens* and *C. tanakaei* (2,457 and 2,195, respectively). However, the distribution of genes into COG categories was not entirely similar in all the four compared genomes. In addition, *E. massiliensis* shared 887, 1,019 and 1,048 orthologous genes with *Coriobacterium glomerans, Collinsella aerofaciens* and *Collinsella tanakaei*, respectively. The average nucleotide sequence identity ranged from 71.38 to 74.08% among *Coriobacteriaceae* family members, and from 72.49 to 74.08% between *E. massiliensis* and other genera (Table 6.).

**Table 6:** ***Number of orthologous genes (upper right), average nucleotide identity levels (lower left) between pairs of genomes ***and numbers of proteins per genome (bold).

	*Enorma massiliensis*	*Collinsella aerofaciens*	*Collinsella tanakaei*	*Coriobacterium glomerans*
*Enorma massiliensis*	**1,901**	1,019	1,048	887
*Collinsella aerofaciens*	74.08	**2,157**	1,041	880
*Collinsella tanakaei*	73.66	74.28	**2,195**	909
*Coriobacterium glomerans*	72.49	71.54	71.38	**1,768**

## Conclusion

On the basis of phenotypic, phylogenetic and genomic analyses (taxono-genomics), we formally propose the creation of *Enorma massiliensis* gen. nov., sp. nov. which to accommodate strain phI^T^. This strain has been cultivated from an obese patient in Marseille, France.

### Description of *Enorma* gen. nov.

*Enorma* (e. nor’ma where strain N.L. fem. N. enorma, from enormis, beyond the norm in Latin, in reference to the overweight status of the patient from whom strain phI^T^ was cultivated).

Gram-positive rods. Strictly anaerobic. Mesophilic. Non motile. Negative for catalase, oxidase, nitrate reduction and indole productions. Positive α-galactosidase, β-galactosidase, arginine dihydrolase, arginine arylamidase, proline arylamidase, histidine arylamidase, α and β-glucosidase, mannose and raffinose fermentation. The habitat of the organism is the human digestive tract. The type species is *Enorma massiliensis*.

### Description of *Enorma massiliensis* sp. nov.

*Enorma massiliensis* (mas.si.li.en′sis. L. masc. adj. *massiliensis* of Massilia, the Roman name of Marseille, France, where type strain phI^T^ was isolated).

Colonies were light grey measuring 0.4 mm in diameter on blood-enriched Columbia agar, they are bright and stained grey Cells are rod-shaped with a mean diameter of 0.57 μm. Optimal growth is achieved under anaerobic conditions with a CO_2_ atmosphere. No growth is observed under aerobic conditions. Growth occurs between 37-45°C, with optimal growth observed at 37°C on blood-enriched Columbia agar. Cells are Gram-positive, non endospore-forming, and non motile. Cells are negative for catalase and oxidase. Negative reactions were observed for nitrate reduction, indole production, alkaline phosphatase and urease, β-galactosidase 6 phosphate, α-arabinosidase, β-glucuronidase, N-acetyl-β-glucosaminidase, glutamic acid decarboxylase, α-fucosidase, leucyl glycine arylamidase, phenylalanine arylamidase, leucine arylamidase, pyroglutamic acid arylamidase, tyrosin arylamidase, alanine arylamidase, glycine arylamidase, glutamyl glutamic acid arylamidase, alanine arylamidase, glycine arylamidase, tyrosine arylamidase and serine arylamidase. Using an API 50CH, fermentation or assimilation was not observed. Positive reactions were observed for α-galactosidase, β-galactosidase, arginine dihydrolase, arginine arylamidase, proline arylamidase, histidine arylamidase, α and β-glucosidase, mannose and raffinose fermentation. Cells are susceptible to amoxicillin, amoxicillin-clavulanic acid, metronidazole, imipenem, vancomycin, nitrofurantoin, rifampicine, gentamycin 500 and resistant to erythromycin, penicillin, doxycyclin, ciprofloxacin, ceftriaxone and trimethoprim/sulfamethoxazole. The 16S rRNA and genome sequences are deposited in Genbank and EMBL under accession numbers JN837493 and CAGZ00000000, respectively. The G+C content of the genome is 62.0%. The habitat of the organism is the human digestive tract. The type strain phI^T^ (= CSUR P183 = DSMZ 25476) was isolated from the fecal flora of an obese French patient. This strain has been found in Marseille, France.
